# The Association of CXC Receptor 4 Mediated Signaling Pathway with Oxaliplatin-Resistant Human Colorectal Cancer Cells

**DOI:** 10.1371/journal.pone.0159927

**Published:** 2016-09-26

**Authors:** Wen-Shih Huang, Meng-Chiao Hsieh, Cheng-Yi Huang, Yi-Hung Kuo, Shui-Yi Tung, Chien-Heng Shen, Yung-Yu Hsieh, Chih-Chuan Teng, Kam-Fai Lee, Te-Chuan Chen, Ko-Chao Lee, Hsing-Chun Kuo

**Affiliations:** 1 Division of Colon and Rectal Surgery, Department of Surgery, Chang Gung Memorial Hospital, Chiayi, Taiwan; 2 Chang Gung University College of Medicine, Taoyuan, Taiwan; 3 Graduate Institute of Clinical Medical Sciences, College of Medicine, Chang Gung University, Chiayi, Taiwan; 4 Department of Hepato-Gastroenterological, Chang Gung Memorial Hospital, Chiayi, Taiwan; 5 Institute of Nursing and Department of Nursing, Chang Gung Institute of Technology Chiayi Campus, Chiayi, Taiwan; 6 Department of Pathology, Chang Gung Memorial Hospital at Chiayi, Taiwan; 7 Division of Nephrology Kaohsiung Chang Gung Memorial Hospital and Chang Gung University College of Medicine, Kaohsiung, Taiwan; 8 Division of Colorectal Surgery, Department of Surgery, Chang Gung Memorial Hospital—Kaohsiung Medical Center and Chang Gung University College of Medicine, Kaohsiung, Taiwan; 9 Chronic Diseases and Health Promotion Research Center, CGUST, Chiayi, Taiwan; 10 Research Center for Industry of Human Ecology, Chang Gung University of Science and Technology, Taoyuan, Taiwan; University of Kansas School of Medicine, UNITED STATES

## Abstract

The stromal cell–derived factor-1 (SDF-1)/CXC receptor 4 (CXCR4) axis plays an important role in tumor angiogenesis and invasiveness in colorectal cancer (CRC) progression. In addition, metastatic CRC remains one of the most difficult human malignancies to treat because of its chemoresistant behavior. However, the mechanism by which correlation occurs between CXCR4 and the clinical response of CRC to chemotherapy remains unknown. We generated chemoresistant cells with increasing doses of oxaliplatin (OXA) and 5-Fluorouracil (5FU) to develop resistance at a clinical dose. We found that the putative markers did not change in the parental cells, but HCT-116/OxR and HCT-116/5-FUR were more aggressive and had higher tumor growth (demonstrated by wound healing, chemotaxis assay, and a nude mice xenograft model) with the use of oxaliplatin. Apoptosis induced by oxaliplatin treatment was significantly decreased in HCT-116/OxR compared to the parental cells. Moreover, HCT-116/OxR cells displayed increased levels of p-gp, p-Akt p-ERK, p-IKBβ, CXCR4, and Bcl-2, but they also significantly inhibited the apoptotic pathways when compared to the parental strain. We evaluated the molecular mechanism governing the signaling pathway associated with anti-apoptosis activity and the aggressive status of chemoresistant cells. Experiments involving specific inhibitors demonstrated that the activation of the pathways associated with CXCR4, ERK1/2 mitogen-activated protein kinase (MAPK), and phosphatidylinositol 3-kinase (PI3K)/Akt is critical to the functioning of the HCT-116/OxR and HCT-116/5-FUR characteristics of chemosensitivity. These findings elucidate the mechanism of CXCR4/PI3K/Akt downstream signaling and provide strategies to inhibit CXCR4 mediated signaling pathway in order to overcome CRC’s resistance to chemotherapy.

## Introduction

Colorectal cancer, one of the most common cancers worldwide, is the leading cause of cancer-related death, but death due to colorectal cancer usually results from uncontrolled metastatic disease [1]. Only a minority of CRC cases are detected at a stage early enough for potential treatment with medicinal therapies such as surgery, chemotherapy, and radiotherapy. Rapid tumor growth is closely linked to chemotherapy resistance [2], which is the major problem affecting CRC therapy; there are no long-term survival rates for CRC because tumor cells develop resistance to cytotoxic drugs. Oxaliplatin (OXA) and 5-Fluorouracil (5FU)-based adjuvant chemotherapy are routinely given to patients with CRC [3]. Nevertheless, chemoresistance is a major obstacle for CRC treatment, and metastatic CRC patients are extensively resistant to chemotherapy [4]. Oxaliplatin is a third-generation platinum-derived chemotherapeutic agent [5]. It covalently binds DNA, forming platinum-DNA adducts that cause cell death, and it appears to block DNA replication. Although the development of resistance to OXA is becoming a problem for successful chemotherapy in the treatment of CRC, few therapies to target remaining pathways have been investigated [6]. Several clinical and experimental studies have shown that a loss of E-cadherin at the cell membrane surface is a key hallmark of epithelial-mesenchymal transition (EMT), and these studies have described a reverse relationship between E-cadherin and drug resistance [7–8]. In addition, EMT occurs widely in tumor cell invasion and metastasis, and P-glycoprotein 1 (P-gp) occurs in the expression of multidrug resistance (MDR) [9]. These observations have led to studies on the potential mechanism for the association between EMT induction and the development of drug resistance, and to discussion concerning new approaches to the clinical management.

In the case of colon cancer treated with surgery, CXCR4 and Bcl-2 protein expression may be a predictor of tumor cell invasion and metastasis[10–11], which are associated with EMT phenotype and creating chemoresistance, processes that involve the activation of the pathways associated with phosphatidylinositol 3-kinase/Akt, extracellular regulated kinase (ERK), and NF-kappaB by tumor cells or by surrounding stromal cells [12–13]. Several clinical and experimental studies have shown that the activation of the Akt/ ERK1/2 pathways directly regulates P-gp, Bcl-2, and CXCR4 in colorectal cancer MDR cells associated with many physiologic and pathologic processes involving inflammation[14–15]. Several studies have indicated that chemokines play a crucial role in tumor cell invasion and metastasis [16]. Cancer cells express different forms of chemokine receptors and those individual ligands are expressed in tissues to which these cancers commonly metastasize. Stromal cell–derived factor (SDF)-1 (CXC chemokine ligand-12), a member of the CXC chemokine family, is expressed in stromal tissues in multiple organs. SDF-1 elicits its effect through its specific CXC chemokine receptor (CXCR4), which is known to play roles in tumor metastasis and chemotherapy resistance [17]. However, the molecular mechanism for the role of the CXCR4 mediated signaling pathway in promoting resistance to OXA-based chemotherapy and how that could be overcome are not known.

Our previous studies have indicated that colorectal cancer cells express CXCR4 and that SDF-1 promotes their survival and migration to distant tissues. We investigated the chemoresistant cell lines and the molecular and phenotypic variations *in vitro* and *in vivo*[14–18]. Although CXCR4 is known to regulate the PI3K/Akt and MAPK cascade, the specific mechanism by which chemoresistance occurs in colorectal cancer cells has not been adequately documented. In the present study, we showed that the expression of CXCR4 and Bcl-2 was upregulated in HCT-116/OxR cells in response to the activation of PI3K/Akt and ERK1/2 intracellular signaling cascades, and the transcription factor NFκB. Our findings provide evidence of the molecular mechanism by which CXCR4 mediated an anti-apoptosis pathway in chemoresistant colorectal cancer cells with OXA, suggesting that CXCR4 signaling is associated with drug resistance.

## Materials and Methods

### Materials

All culture materials were purchased from Gibco (Grand Island, NY, USA). 3-(4,5-dimethylthiazol-2-yl)-2,5-diphenyltetrazolium bromide (MTT), ERK inhibitor (PD98059), Akt inhibitor **(**LY294002), and NF-κB inhibitor (Pyrrolidine dithiocarbamate (PDTC)) were purchased from Sigma (St. Louis, MO, USA). Mouse monoclonal antibodies against p-gp, p-IKBβ, CXCR4 and Bcl-2, and β-actin were purchased from Santa Cruz Biotechnology (Santa Cruz, CA, USA). Rabbit polyclonal antibodies against ERK1/2Thr^202^Ty^r204^, p38 Thr^180^Tyr^182^, and Akt Thr^308^ mouse monoclonal cdk1 antibody were purchased from Cell Signaling Technology (Beverly, MA, USA). The TdT-mediated dUTP Nick End Labeling (TUNEL) kits were from Roche (Germany). SDS, NP-40, sodium deoxycholate, protease inhibitor cocktails were purchased from Sigma (St. Louis, MO, USA). AMD3100 (CXCR4 inhibitor), and all other chemicals of reagent grade were obtained from Sigma (St Louis, MO, USA).

### Development of chemoresistance cell lines

The colon cancer HCT-116 cell line was purchased from the Bioresources Collection and Research Center (BCRC) of the Food Industry Research and Development Institute (Hsinchu, Taiwan). Cells were maintained in Dulbecco’s Modified Eagle Medium (DMEM) supplemented with 10% fetal bovine serum (FBS) and 1% penicillin/streptomycin in a CO_2_ incubator at 37°C. The oxaliplatin and 5FU resistant cell line were developed as described previously [19–20]. Cells solidly resistant to Oxaliplatin or 5FU were established by exposing parental HCT-116 cells to an initial dose of 0.1 μg/mL and culturing alive cells to a confluence of 80% for three passages (about 6 weeks). The cells that survived initial Oxaliplatin and 5FU treatment were treated to 0.5 μg/mL for the same three passages (about 6 weeks and then 1.0 μg/mL for another three passages (about 6 weeks). Finally, the Oxaliplatin and 5FU concentration was increased to the concentration of 2 μg/mL for 3 weeks (at 10 weeks). The chemotherapy resistance cells were named HCT-116/OxR and HCT-116/5-FUR, which were cultured in DMEM supplemented with 10% fetal bovine serum (FBS), penicillin-streptomycin. Chemoresistance cells were continuously cultured in the concentration of 2 μg/mL of the respective drugs, unless otherwise indicated [20].

### Cell growth and proliferation assay

The previously reported MTT quantitative colorimetric assay was verified as being capable of detecting viable cells and was used for cell viability determinations as previously described [21]. Cells were seeded and incubated with the various agents. Thereafter, the medium was changed, and cells were incubated with MTT (0.5 mg/mL) for 4 h. The viable cell number was directly proportional to the production of formazan, which was measured spectrophotometrically (λ = 563 nm) after solubilization with isopropanol. Cell growth was determined by counting the cells at the indicated time points with a Coulter counter, combined with a trypan blue (0.2%) exclusion assay.

### Apoptosis assay and cell cycle distribution analysis

Changes in cell morphological characteristics during apoptosis were examined using fluorescence microscopy of 4′,6-diamidino-2-phenylindole (DAPI)-stained cells. The monolayer of cells was fixed with 4% paraformaldehyde for 30 min at room temperature. The fixed cells were permeabilized with 3 treatments in 0.2% Triton X-100 in phosphate-buffered saline, followed by incubation with 1 μg/mL of DAPI for 30 min. The apoptotic nuclei were detected under 200× magnification using a fluorescent microscope with a 340/380 nm excitation filter and were scored according to the percentage of apoptotic nuclei found in samples containing 200 to 300 cells. Cell-cycle distribution was analyzed using flow cytometry. Cells stained with propidium iodide were analyzed with a FACScalibur™ (Becton Dickinson), and the data were analyzed using a mod-fit cell cycle analysis program [22].

### Preparation of total cell extracts and immunoblot analyses

Cells were lysed with a buffer containing 1% NP-40, 0.5% sodium deoxycholate, 0.1% sodium dodecyl sulfate (SDS), and a protease inhibitor mixture (phenylmethylsulfonyl fluoride, aprotinin, and sodium orthovanadate). The total cell lysate (50 μg of protein) was separated by SDS-polyacrylamide gel electrophoresis (PAGE) (12% running, 4% stacking) and analyzed by using the designated antibodies and the Western-Light chemiluminescent detection system (Bio-Rad, Hercules, CA),. The area of the photo images in immunoblot was determined by measuring the numbers of pixels using by ImageGauge 3.46 software (Fujifilm, Inc.) as previously described [23]. Quantitative analysis of fluorescence assays using ImageJ, images are captured onto the hard drive of the workstation computer [24].

### *In vivo* treatment

BALB/c-*nu* mice were purchased from the National Laboratory Animal Center in Taiwan, and kept individually in a 12-hour light/dark cycle cage and had free access to water and food. Animal care and the general protocols for animal use were approved by the Institutional Animal Care and Use Committee of Chang Gung University of Science and Technology. BALB/c-*nu* female nude mice, 4–6 weeks old (18–20 g), were maintained under Specific Pathogen Free (SPF) conditions and supplied with sterilized food and water. After trypsinizing, the HCT-116/OxR and parental cells (10^6^ cells/0.2 ml) were injected subcutaneously into the flanks of female athymic BALB/c-*nu* mice (4–6 weeks old). After tumor inoculation, the mice were divided randomly into four groups of eight mice each. The control animals were treated daily with 0.1 mL DMSO (0.25%; i.p.), and the test animals were treated with Oxaliplatin (5 mg/Kg; i.p.) for five days. Tumor volumes were monitored on the fourth day, and then measured at four-day intervals using calipers; calculation was based on the following formula: length × width^2^ × π/6 [25–26]. To monitor drug toxicity levels, the body weights of the mice were measured every week. After 18 days, the mice were euthanized, and the tumors were removed and assayed. In addition, a pathologist examined the organs of each mouse, including the liver, lungs, and kidneys [21].

### Histochemistry and immunohistochemistry

Tumor tissue sections were fixed in formaldehyde and embedded in paraffin blocks. The slides were stained with hematoxylin and eosin, and mounted for microscopic examination. For immunohistochemical analysis, 5 μm thick sections from each subcutaneous tumor specimen were fixed and incubated with monoclonal anti-p-gp, CD133, CXCR4 and Bcl-2 antibodies (Santa Cruz, CA, USA) and then with 1:100 diluted biotinylated horse anti-mouse IgG for 1 h. After being washing with PBS, they were reacted with 1:100 diluted avidin-biotin peroxidase mixture (Vectastain Universal Elite ABC Kit) for 30 min. The sections were washed thoroughly in PBS, and slides were counterstained with hematoxylin. Finally, the slides were washed, dehydrated, and mounted for microscopic examination. The immunoreative cells (brown) were counted. The digital images were captured using a digital camera (Canon A640). The positive area and optical density (OD) of positive cells + (brown) were determined by measuring three randomly selected microscopic fields (400× magnification) for each slide. The IHC index was defined as average integral optical density (AIOD) (AIOD = positive area×OD/total area) [25].

### Statistical analysis

The experiments were performed in triplicate independent experiments, and data were presented as three repeats from one independent experiment. Data are reported as the mean ± standard deviation and evaluated by one-way ANOVA. Significant differences were established at *P* < 0.05 [26].

## Results

### Establishment of chemoresistant cell lines

**Fig 1:**
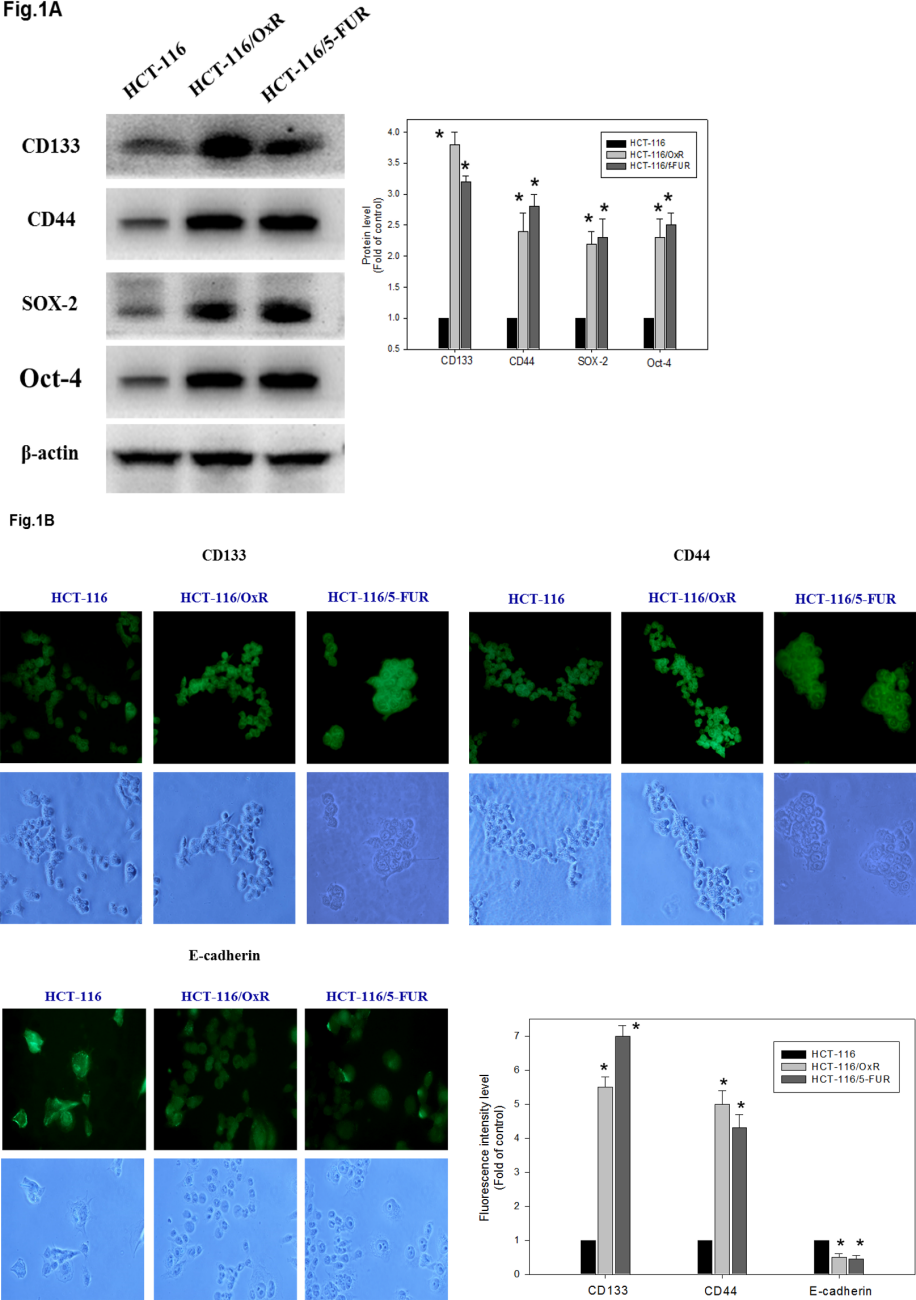
Expression of markers CD133, CD44, SOX-2 and OCT-4 in parental cells of HCT-116 and chemoresistant cell lines of HCT-116/OxR and HCT-116/5FUR. (A) Western blotting showed that CD133, CD44, SOX-2 and OCT-4 proteins were enriched expressed in the chemoresistant cells compared with parental cell. Protein levels were quantified by densitometric analysis with the control being set at 100%. Data presented in western blot are derived from a representative study, and comparisons of protein expression are calculated from three independent experiments. *P < 0.05, when compared with control HCT-116 group. (B) Immunofluorescence analysis showed expression of markers CD133, CD44 and E-cadherin in parental cell line of HCT-116 and chemoresistant cell line of HCT-116/OxR and HCT-116/5-FUR.

To establish stable colon cancer cell lines continuously resistant to OXA or 5FU, HCT-116 cells were exposed to increasing concentrations of OXA or 5FU [19]. Chemoresistant cells displayed elongated and dyscohesive features. These cells are referred to as having the characteristics of tumor-initiating cells, tumor-promoting cells, or cancer stem cells (CSC). The expression profiles of parental HCT-116 human CRC cells and cells resistant to OXA or 5FU (HCT-116/OxR or HCT-116/5FUR) were evaluated by western blot and immunofluorescence stain. HCT-116/OxR and HCT-116/5FUR cells expressed significantly more CD133 and CD44, putative CSC markers as well as SOX2 and OCT4 [27] than did parental HCT-116 cells (Fig 1). Transcription factors for maintaining the survival of cancer stem cell–like cells and anti-apoptotic activity were found in colorectal cancers.

### Effect of HCT-116/OxR on OXA-inhibited tumor growth, migration, and invasion *in vitro* and *in vivo*

**Fig 2:**
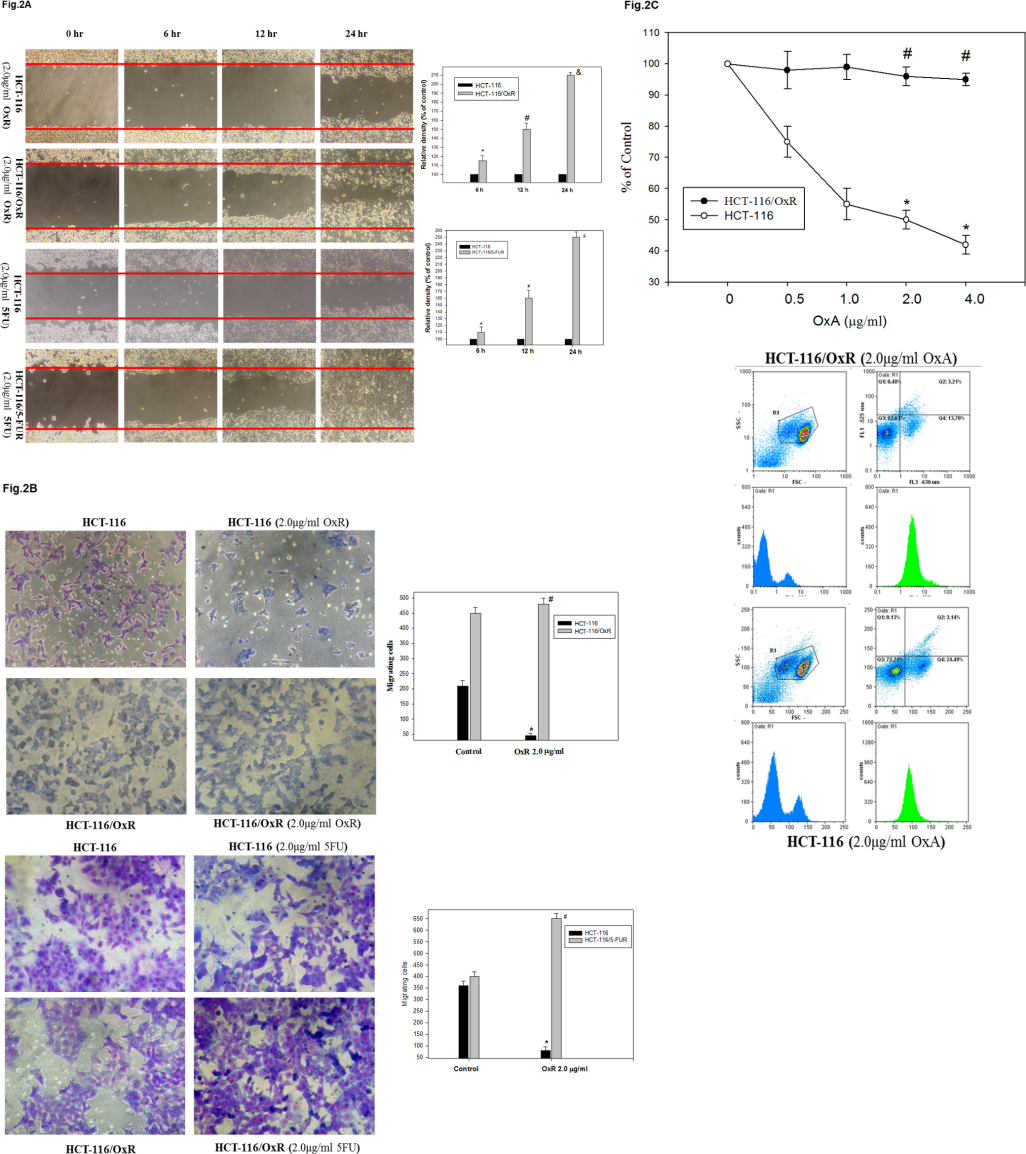
Representative chemosensitivity showed wound healing assay and assessment of cell death on HCT-116/OxR and HCT-116/5-FUR cells depending on the concentration of OxA and 5-FU. (A) HCT-116, HCT-116/OxR and HCT-116/5-FUR cells were incubated with OxA and 5-FU for 6, 12 and 24 h, and the migration using the scratch-wound assay was visualized as described in Methods. The percentage of surface area filled by the parental HCT-116 CRC cell line was subsequently quantified by densitometric analyses relative to that of the control, which was set at 100% as shown in the graph. The variable *, #, & symbols indicate means that are significantly different when compared to the control group of different times incubation with *P* < 0.05, respectively. (B) Determination of the effects of OxA and 5-FU treatment on cell migration and invasiveness cells. Invasion through a layer of Matrigel was determined by a Boyden Chamber method as described in Methods. The lower and upper chemotaxis cells were separated by a polycarbonate membrane. Microscopy images detected cells that migrated into the inner membrane. Magnification: × 200. The cell migration was quantified by counting the number of cells that migrated into the inner membrane. Control cells remained untreated. The invasiveness was quantified and is presented in the graph. (C) Treatment for 24 h, the cells were stained with FITC-conjugated Annexin-V and PI for flow cytometry analysis as described in Materials and Methods. P < 0.01, compared with the control group. *p < 0.05, cell death assays, untreated group versus OxA treated HCT-116 group. #P < 0.05, untreated group versus OxA treated HCT-116/OxR group.

**Fig 3:**
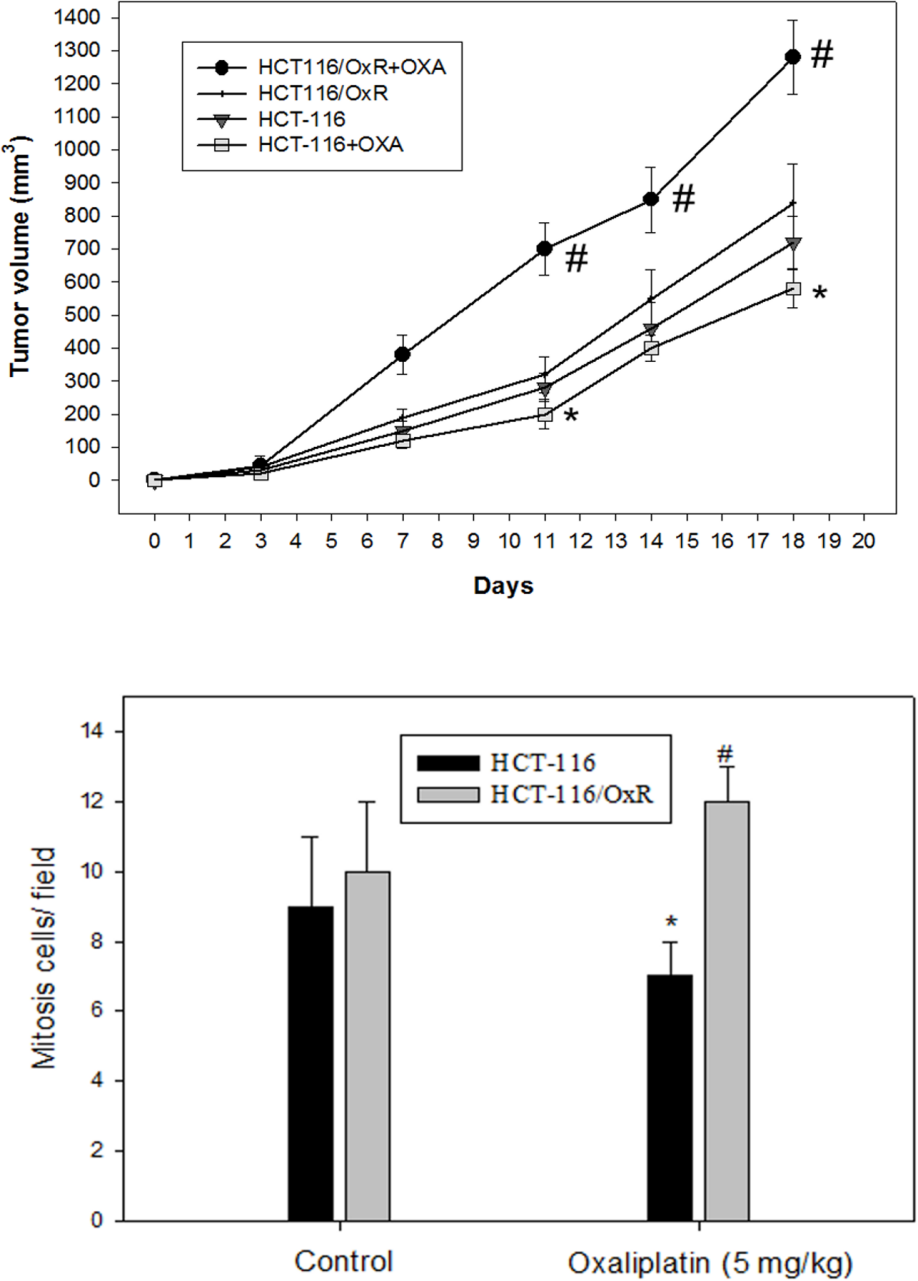
Growth inhibition of HCT-116 and HCT-116/OxR cells xenograft by oxaliplatin. Nude mice were implanted subcutaneously with cells into flanks on day 0, then treated with or without (as a control) oxaliplatin. (A) Time courses effect of oxaliplatin on the growth of cells xenograft was evaluated by determining the tumor volume every four days. (B) On the 24th day after tumor implantation, the tumor of each mouse was removed and stained with hematoxylin–eosin. The mitosis cells were determined by microscopic examination and counted from 10 fields (200 X magnification) of each tumor sample. Statistical analysis results are the means ± SEM of mitosis cells per microscope field from eight animals per group (n = 8; per group). *P < 0.01, compared with the HCT-116 control group; #P < 0.01, compared with the HCT-116/OxR control group.

**Fig 4:**
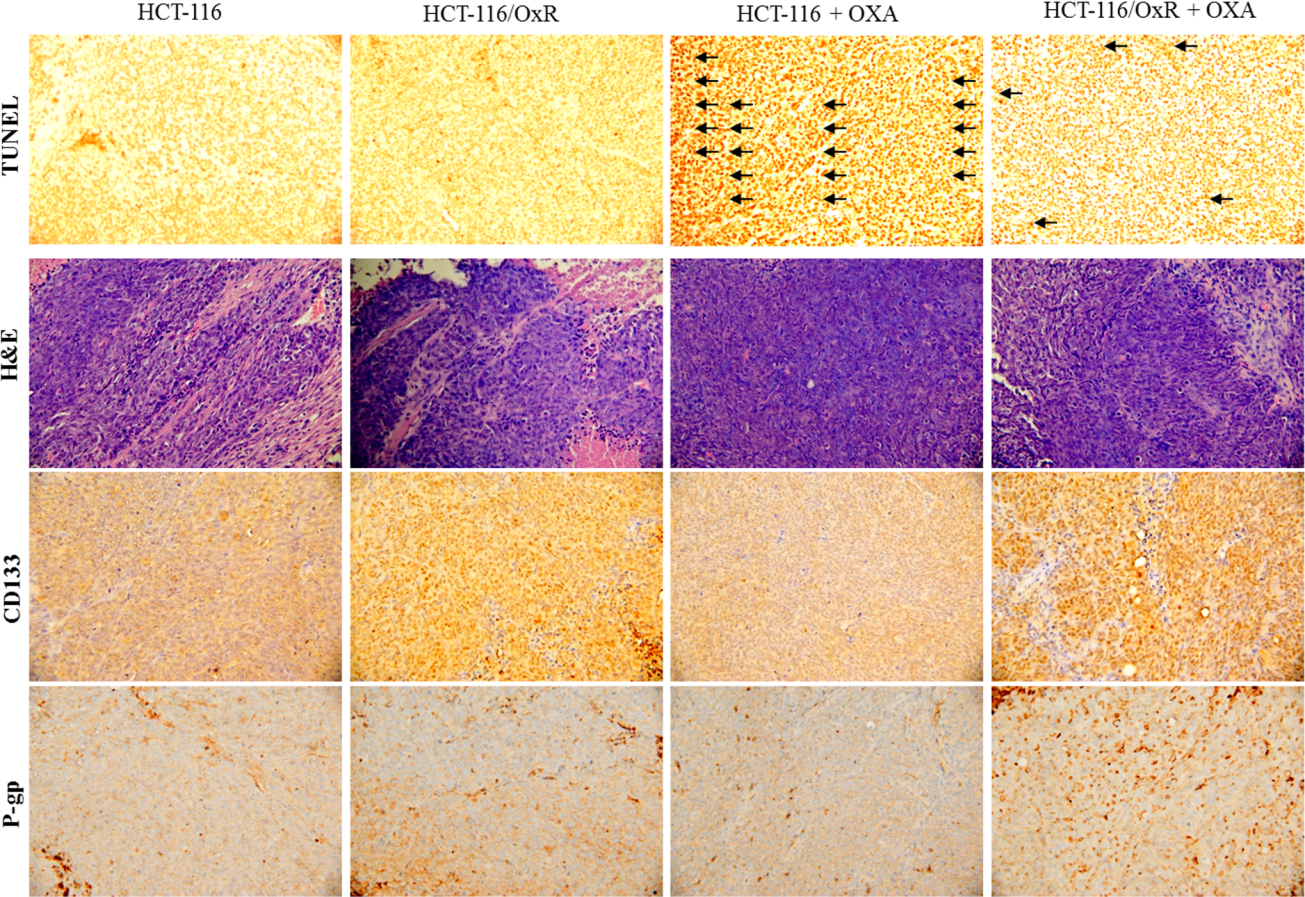
*In vivo* tumor characteristics of chemosensitivity on cells xenograft by oxaliplatin. HCT-116 and HCT-116/OxR cells removed and the section were stained by immunohistochemical analysis as described in text. Immunohistochemical analysis of tumors was conducted, and multiple tumor fields were evaluated per group. Representative images for all groups from both experiments are presented. TUNEL staining revealed significantly greater apoptosis in tumors. H&E staining revealed similar s.c. tumor morphology among all groups of tumors. CD133 and P-gp staining showed expressed tumor cells treated with oxaliplatin.

Using the scratch-wound assay, a lasting rapid movement was observed in resistant cells. A resultant movement of an HCT-116 cell migration front was clearly evident at 24 h, where a highly confluent (90%–100%) monolayer region gradually moved into the cell-free scratch region. In the presence of OXA and 5FU, at a clinically relevant plasma concentration, migration in HCT-116/OxR and HCT-116/5-FUR were highly increased after 12 h and 24 h of incubation, whereas OXA and 5-FU led to a virtually complete inhibition of parental HCT-116 cell proliferation and sensitivity to drugs (Fig 2A). The Boyden chamber assay was used to evaluate the *in vitro* migration and invasion effect of treatment with antineoplastic drugs associated with the aggressive status of HCT-116, HCT-116/OxR and HCT-116/5-FUR cells. The observations revealed that OXA and 5-FU resulted in a remarkable inhibition of parental HCT-116 cell migration as compared to no treatment alone, but HCT-116/OxR and HCT-116/5-FUR cells showed highly evident movement and invasive properties (Fig 2B). To quantify the anti-apoptosis activity of OXA upon resistant cells, we subjected HCT-116 and HCT-116/OxR cells to a dose range of OXA for 24 h and performed viability assays. The half-maximal inhibitory concentration (IC50) of an OXA treatment upon proliferation was sensitively 2.0 μg/ml in HCT-116 cells. HCT-116/OxR cells were resistant to OXA as expected with 95% of viable cells. Similarly, as expected, oxaliplatin-resistant cells were resistant to oxaliplatin as determined by annexin V-FITC/PI dye (Fig 2C). In addition, to determine the *in vivo* effects of OXA on tumor-growth resistance in HCT-116/OxR cells, we exposed human parental HCT-116 and HCT-116/OxR cells to indicated OXA doses for 18 days and examined them by assay of xenografted tumor in nude mice, as described previously. The results of previous toxicity-assessment experiments suggested that the *in vivo* dosage of OXA should not exceed 5 mg/kg/day; therefore, daily intraperitoneal injections of OXA were used in these experiments. Fig 3 shows the time course of xenograft growth with and without OXA treatment. Evaluation of the xenograft HCT-116 tumor volume showed that a significant inhibition of tumor growth by OXA was caused in a time-dependent manner. However, the HCT-116/OxR xenograft volumes were reduced 2.5 fold more than parental HCT-116, which was the control group (P < 0.05). At the end of this experiment, the xenograft tumors were removed. Evaluation of a proliferative mitosis by H&E staining of the tumor sections showed that OXA led to an increase in the number of proliferating HCT-116/OxR cells in tumors compared with those untreated in the control group. Fig 4 shows the representative photo images from the tumor sections.

### Effect of OXA-resistant cells on *in vivo* cellular phenotype and anti-apoptosis status

We undertook to verify the effects of oxaliplatin treatment on the relationship between apoptosis and the aggressive status in tumors derived from parental and chemoresistant cells that were treated with OXA (Fig 4). Quantification of TUNEL staining showed that OXA treatment resulted in significantly more apoptosis in tumors derived from parental HCT-116 cells than in tumors from HCT-116/OxR cells (HCT-116/OxR group = 28±4; HCT-116 group = 121±5, *p*<0.05). Based on these studies, by the microscopic examination of the nuclei or cytoplasmic morphology of tumor sections, we tested whether resistant cells with OXA had an effect on cell proliferation *in vivo*. We found that the proliferating cells of the HCT-116/OxR+OXA group showed abundant mitosis (Fig 4). We then used immunohistochemistry to examine another marker of resistant cells’ aggressive status, the expression of CD133 and P-gp, both of which were more upregulated in the HCT-116/OxR groups than the HCT-116 groups with or without OXA. These results indicated the chemoresistant actions of OXA on HCT-116/OxR cells growing *in vivo*. To further examine the signaling pathways associated with anti-apoptosis and the aggressive status of chemoresistant cells, we studied the effects of kinase inhibitor on resistance to OXA-induced apoptosis and the less aggressive situation in HCT-116/OxR cells. Using the apoptosis, scratch-wound assay and boyden chamber assay, cells were followed to OXA and then co-treated with inhibitors, including CXCR4 inhibitor AMD3100 (100 nM), PI3K/AKT inhibitor LY294002 (10 μM), ERK1/2 inhibitor PD98059 (30 μM), and NF-kappaB inhibitor PDTC (30 μM). As shown in Table 1, these treatments significantly blocked the chemoresistance of OXA at 35%, 25%, 30%, and 28%, respectively (*P*< 0.01), and then decreased the aggressive status of the HCT-116/OxR cells, respectively (*P*< 0.01). The results from these experiments confirmed that pretreatment of HCT-116/OxR cells with CXCR4 inhibitor significantly inhibited the OXA resistance of HCT-116/OxR cells as well as the molecular mechanism by which CXCR4 contributes to OXA resistance of CRC associated with the MAPK- or PI3K/Akt-dependent pathways.

**Table 1:** Effects of the kinase inhibitor on the oxaliplatin treatment associated with apoptosis and aggressive status in HCT-116 and HCT-116/OxR cells.

	% of cell death	Migration (%)	Cell invasion (%)
**HCT116+OXA**	**32**	**100**	**100**
**HCT116/OxR+OXA**	**14**	**210**	**980**
**HCT116/OxR**	**35**	**92**	**250**
**OXA+AMD**			
**HCT116/OxR**	**25**	**120**	**380**
**OXA+LY**			
**HCT116/OxR**	**30**	**131**	**430**
**OXA+PD**			
**HCT116/OxR**	**28**	**135**	**360**
**OXA+PDTC**			

### PI3K/Akt and ERK1/2 pathways mediate OXA resistance of HCT-116/OXR cells

**Fig 5:**
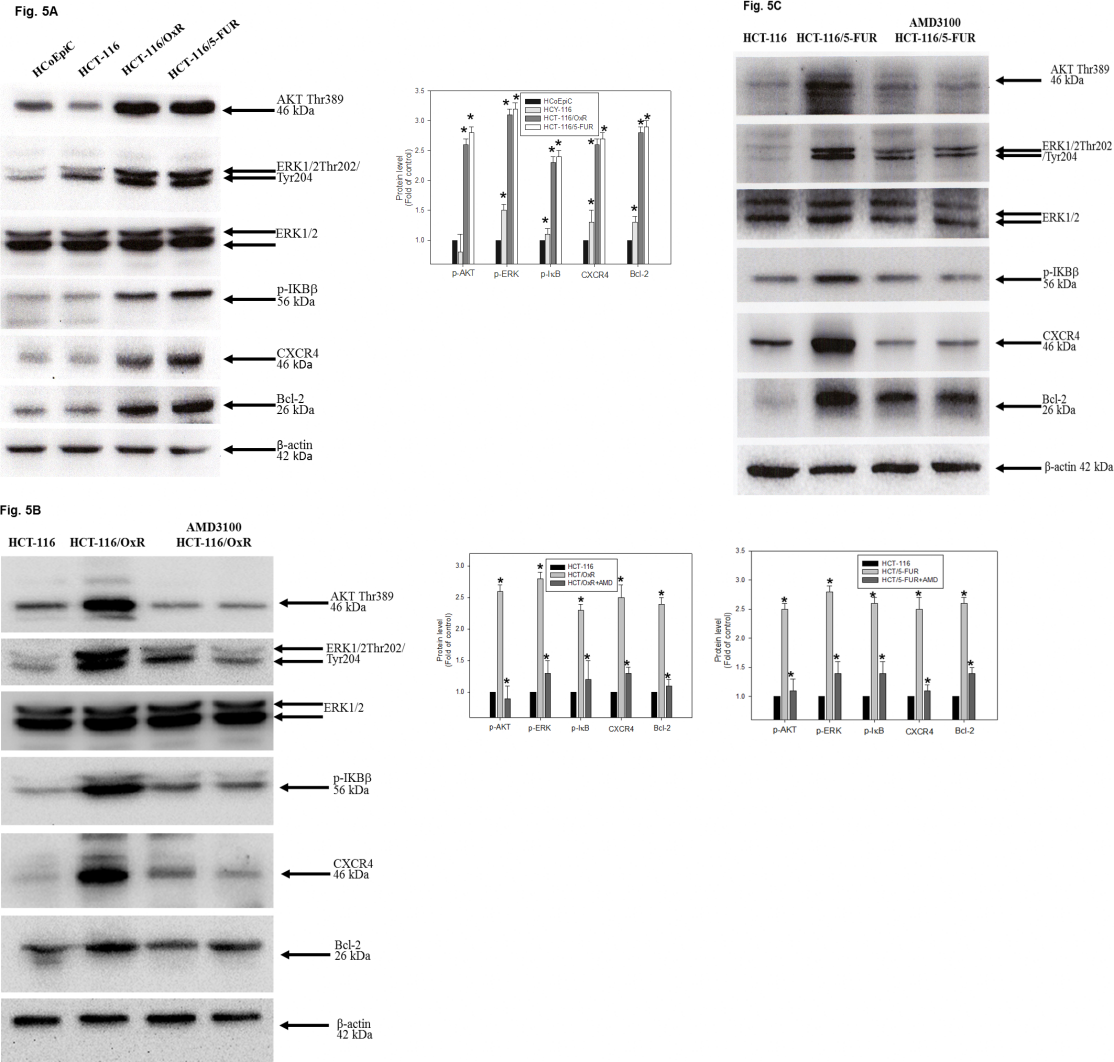
Effect of CXCR4 inhibition on AKT, ERK1/2 and NF-kB pathways. (A) Whole cell lysate proteins were prepared and analyzed by Immunoblotting, using antisera to detect Akt, ERK, IKBβ signaling as well as CXCR4 and Bcl-2. *P < 0.05, when compared with control HCoEpiC group. Whole cell lysate proteins were prepared and analyzed by western blot, with β-actin serving as loading control. Data presented in western blot are derived from a representative study, and comparisons of protein expression are calculated from three independent experiments. (B)(C) HCT-116, HCT-116/OxR and HCT-116/5-FUR cells were pretreated with or without AMD3100. *P < 0.05, when compared with untreated control HCT-116 group.

**Fig 6:**
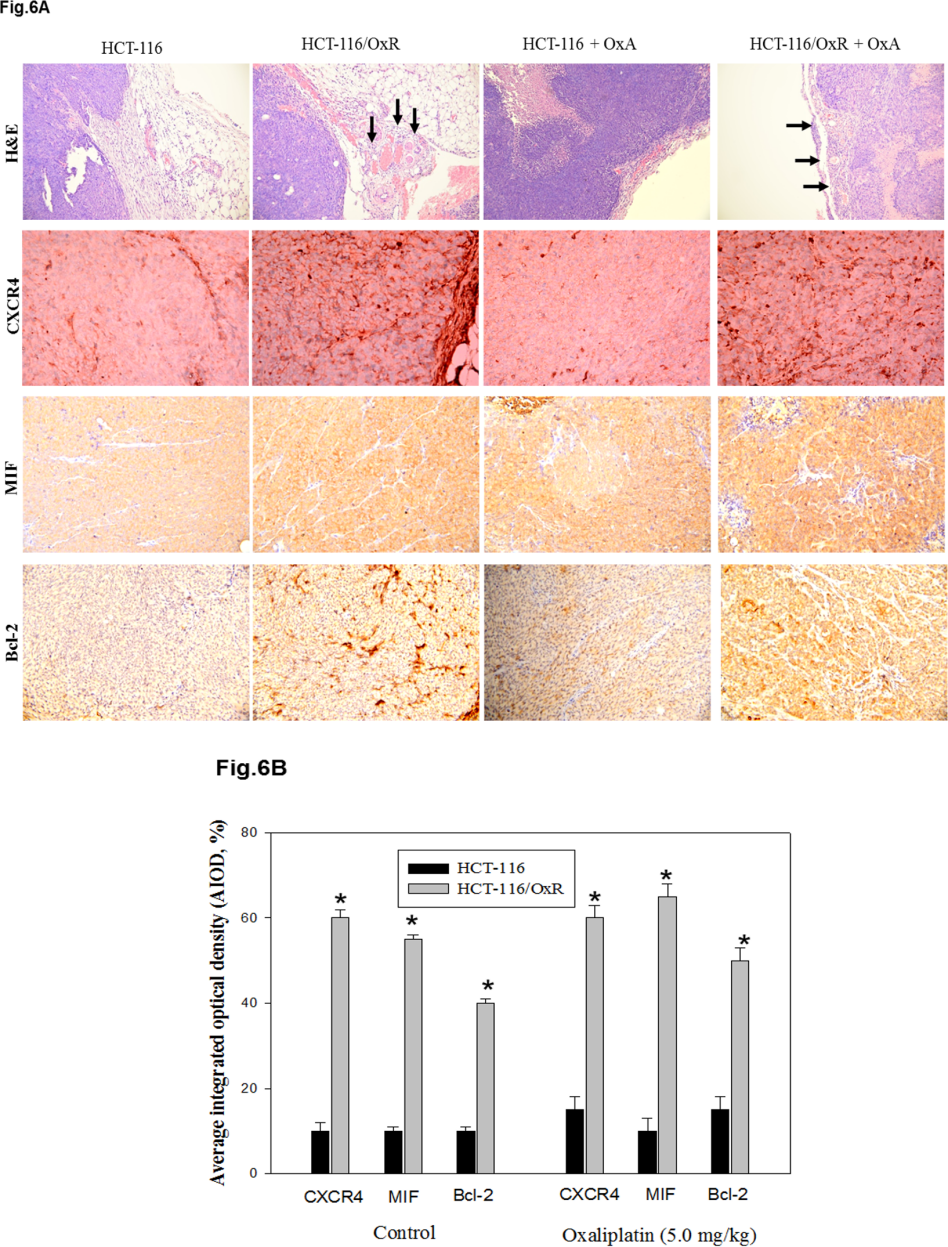
Effect of chemoresistance on *in vivo* MIF-CXCR4 axis and Bcl-2 expression. (A) Immunohistochemical staining for indicated proteins expression *in vivo* tumor aggressive phenotype. H&E staining revealed tumor growth among all groups of vessel numbers. (B) The positive stained area was evaluated from three randomly selected observation fields of each sections. Quantitative of immunohistochemical proteins C-X-C chemokine receptor type 4 (CXCR-4), MIF and Bcl-2 by average integrated optical density (AIOD). Positive stained area was evaluated from three randomly selected observation fields of each section. Data were expressed as means ± SEM (n = 8/group). * p < 0.05, compared with control group, parent group. Magnification ×400.

To gain insight into the molecular mechanism by which CXCR4 is involved in the chemoresistance of CRC, we checked the expression of invasion and anti-apoptosis-related proteins. We observed significantly increased expression of CXCR4 and Bcl-2 as well as Akt, ERK1/2, and IκBβ phosphorylation in the HCT-116/OxR and HCT-116/5-FUR cell lines than human colonic epithelial cells (HCoEpiC), which are widely used as a model for human normal colon cells in studies of cell signaling and apoptosis (Fig 5A). By the way, this was examined to elucidate the mechanism of CXCR4-mediated PI3K/Akt and ERK1/2 signaling proliferation and anti-apoptosis activity. In both cell lines, HCT-116/OxR and HCT-116/5-FUR cells were incubated with the specific inhibitors of CXCR4 (AMD3100) for 1 h while downregulating Akt, ERK1/2, and IκBβ phosphorylation as well as CXCR4 and Bcl-2 expression (Fig 5B and 5C). HCT-116/5-FUR and HCT-116/OxR chemoresistant cells were mediated by CXCR4 mediated signaling pathway. Interestingly, recent studies have indicated the pivotal role of CXCL12/CXCR4 expression in tumor metastasis, showing that it is actively implicated in angiogenesis. In addition, previous study has shown that the macrophage migration-inhibitory factor (MIF), MIF-CXCR4 axis is important in drug resistant colon cancer cells as the critical autocrine CXCR4 ligand [28]. To investigate the *in vivo* role of chemoresistance in CXCR4-mediated tumor vascularization and existential expression, a basic study was conducted to evaluate the angiogenic factors reflected in the aggressiveness of tumor cells by H&E staining of tumor sections. As shown in Fig 6, photographs of tumor vascularization revealed that vessel numbers around HCT-116/OxR cells increased with or without OXA treatment in mice. We also investigated highly the immunochemical expression of CXCR4, MIF and Bcl-2 in HCT-116/OxR tumor sections. This has been validated by quantitative evaluation, showing protein intensity by the average integral optical density. The possible contribution of the MIF-CXCR4 axis in the proliferation of drug-resistant cells in the invasive phenotype of HCT-116/OxR cells will be determined further. Taken together, the results showed that CXCR4 expression in OXA-resistant cells is essential to involvement in PI3K/Akt and ERK1/2 signaling mediated cell death and metastasis.

## Discussion

Oxaliplatin is the most widely used first platinum-based drug for killing colorectal cancer cells. Used in a third-generation platinum-compound-based chemotherapy in combination with 5-fluorouracil and leucovorin (FOLFOX), oxaliplatin improves patient survival [29]. Unfortunately, resistance remains a significant obstacle to its clinical success. Therefore, the identification of the mechanism of oxaliplatin resistance is required to further improve the efficacy of OXA. Many investigators have revealed the mechanism of OXA resistance is still unclear [30]. In this study, we established an oxaliplatin-resistant cell line, HCT-116/OXR, and evaluated the biological characters and resistance mechanism [20]. Interestingly, we determined that oxaliplatin-resistant cells were significantly enriched by the CSC markers CD133 and CD44 as well as SOX-2 and OCT-4 (Fig 1). These OXA resistance cells were exposed to OXA agent that are very likely CSC phenotype [19] and then chemoresistant cells also showed more features associated with enhancing migratory and invasive potential compared to parental cells *in vitro* (Fig 2). Furthermore, HCT-116/OXR cells demonstrated an increased ability to show greater tumorigenicity and an increased ability to prevent OXA-induced apoptosis in subcutaneous tumors from xenografts containing HCT-116/OXR cells of nude mice (Fig 3). HCT-116/OXR cells showed increased *in vivo* resistance to OXA, and this study likewise demonstrated that intraperitoneal injections of OXA (5 mg/kg/day) treatment significantly increased the volume and the number of mitotic cells while maintaining a consistent expression of CD133 and P-gp (Fig 4). More recent studies have also defined transcription factors DCLK1 and LGR5 to play a significant role in quiescent and active stem cells in colorectal cancer. However, we have shown that DCLK1 and LGR5 do not mark in HCT-116/OxR chemoresistant cells with or without OXA treatment in mice in tumor sections by immunochemical evaluation in this study.

It has been previously reported that epithelial-mesenchymal transition (EMT), which modulates cancer progression and metastasis, has been involved in chemoresistance [31]. In keeping with this study, previous reports have identified CSCs in colon solid tumors based on other surface markers such as CD133, CD44, P-gp and pro-survival Bcl-2 protein as well as the EMT phenotype [19, 27]. Our study demonstrated that in an altered phenotype, cells dispersed, developing a spindle shape, in a fashion similar to EMT (Fig 2). Based on the above observations, we hypothesized the effects of molecular loss E-cadherin changes consistent with EMT on the HCT-116/OXR [9]. This possibly played an important role in expressing aggressive phenotypes, and anti-apoptosis status was related to the EMT phenotype. Studies have shown that the mechanism of chemoresistance from HCT-116/OXR undergoes epithelial to mesenchymal or mesenchymal-like transition. This reflects an important process by which cancer cells may have the capability for cancer development through potentially acquiring chemoresistance that induces the activation of related signaling pathways, leading to the inhibition of cell death [31, 32]. Additionally, the PI3K/Akt/ERK1/2/NFκB pathway is directly induced to contribute to CSC biology, including chemoresistance. This shows that therapeutics targeting has demonstrated induction CRC progression and metastasis in malignant cells [33]. The findings indicated that the effect of OXA resistance resulted in migration and invasion ability and that the anti-apoptosis status was mediated by the activation in the expression of Akt and MAPK ERK1/2, and by the upregulation of nuclear NFκB p50 and phosphor IKBβ (Table 1, Fig 5).

Previous studies, largely focused on the expression of CXCR4 on human colorectal cancer cells, suggested that the regulation of the CXCL12/CXCR4 axis in colorectal cell migration, proliferation, and invasion correlated with poor-prognosis angiogenesis [34]; however, the mechanism by which CXCR4 affects Bcl-2 expression in OXA-resistant cells remains unclear. CXCL12-dependent proliferation correlated with the activation of the ERK1/2 and AKT pathways [35]. It have shown that these pathways were involved with the transduction of proliferative signals in tumor cells [11]. Moreover, blocking CXCR4 expression on the cell surface greatly inhibited the ability of colon cancer cells to metastasize to organs [36]. It may have also mediated cancer stem cell trafficking that highly expressed CXCL12. In this study, we aimed to explore the mechanisms of drug resistance and activation of the PI3K/Akt/ERK1/2/NFκB pathways in OXA-resistant cells. In addition, we suggest that the upregulation of CXCR4 and Bcl-2 protein had an effect on OXA resistance, which was preceded by the PI3K/Akt/ERK1/2/NFκB pathway (Fig 5). Using a chemopreventive agent may lead to the specific enrichment of CXCR4-expressing chemoresistant colorectal cells, as shown by an increase in angiogenesis *in vivo* as evidenced by vessel numbers (Fig 6). CXCL12/CXCR4 axis plays a crucial role in determining the homing of metastatic tumor cells with poor prognosis, which the expression of CXCR4 and Bcl-2 and PI3K/Akt and ERK1/2 signaling are controlled and the level at which it is expressed mark the difference between normal and pathological events [37]. Based on our results, it makes possible that in OXA and 5FU resistance colorectal cancer, CXCR4 mediated pathway-related proteins may be studied as potential markers in colorectal cancer samples. Further investigation is required to better understand whether CXCL12/CXCR4 pathway-related proteins can be sufficiently useful in clinical diagnostics in terms of the prediction of response to treatment or prognosis.

In this study, we determined that when cells are lastingly exposed to OXA, they acquire a molecular expression characteristic of the CSC phenotype. Our results imply that CXCR4 mediated an anti-apoptosis and aggressive pathways in chemoresistant colorectal cancer cells with the OXA drug through PI3K/Akt/ERK1/2/NFκB.
